# Water Deficit Diagnosis of Winter Wheat Based on Thermal Infrared Imaging

**DOI:** 10.3390/plants13030361

**Published:** 2024-01-25

**Authors:** Shouchen Ma, Saisai Liu, Zhenhao Gao, Xinsheng Wang, Shoutian Ma, Shengfeng Wang

**Affiliations:** 1 Institute of Quantitative Remote Sensing & Smart Agriculture, School of Surveying and Land Information Engineering, Henan Polytechnic University, Jiaozuo 454000, China; mashouchen@126.com (S.M.); akou185@126.com (S.L.); hygge010309@163.com (Z.G.); hpuwxs@126.com (X.W.); 2 Key Lab for Crop Water Requirement and Regulation of Ministry of Agriculture, Institute of Farmland Irrigation, Chinese Academy of Agricultural Sciences (CAAS), Xinxiang 453002, China; 3 Institute of Western Agriculture, Chinese Academy of Agricultural Sciences, Changji 831100, China; 4 Field Observation and Research Station of Efficient Water Use for Agriculture, Xinxiang 453002, China; 5 School of Water Conservancy, North China University of Water Resources and Electric Power, Zhengzhou 450046, China

**Keywords:** crop canopy temperature, infrared thermography, winter wheat, CWSI, water deficit diagnosis

## Abstract

Field experiments were conducted to analyze the effectiveness of the crop stress index (CWSI) obtained by infrared thermal imaging to indicate crop water status, and to determine the appropriate CWSI threshold range for wheat at different growth stages. The results showed that the sensitivity of plant physiological parameters to soil water was different at different growth stages. The sensitivity of stomatal conductance (Gs) and transpiration rate (Tr) to soil water was higher than that of leaf relative water content (LRWC) and photosynthetic rate (Pn). The characteristics of plant physiology and biomass (yield) at each growth stage showed that the plant production would not suffer from drought stress as long as the soil water content (SWC) was maintained above 57.0% of the field water capacity (FWC) during the jointing stage, 63.0% of the FWC during the flowering stage and 60.0% of the FWC during the filling stage. Correlation analysis showed that the correlation of CWSI with Gs, Tr and Pn was lower than that with LRWC and SWC at the jointing stage. CWSI was extremely significantly negatively correlated with SWC and LRWC (*p* < 0.01), but significantly negatively correlated with Gs, Tr and Pn (*p* < 0.05). At the flowering stage, CWSI was extremely significantly negatively correlated with all physiological and soil parameters (*p* < 0.01). The regression analysis showed that the CWSI of winter wheat was correlated with biomass (grain yield) in a curvilinear relationship at each growth stage. When the CWSI increased to a certain extent, the biomass and yield showed a decreasing trend with the increase in CWSI. Comprehensive analysis of all indexes showed that CWSI can be used as a decision-making index to guide the water-saving irrigation of winter wheat, as long as the CWSI threshold of plants was maintained at 0.26–0.38 during the jointing stage, 0.27–0.32 during the flowering stage and 0.30–0.36 during the filling stage, which could not only avoid the adverse effects of water stress on crop production, but also achieve the purpose of water saving.

## 1. Introduction

Winter wheat is the main food crop in the Huang–Huai–Hai region of China. In recent years, the water-saving irrigation technology of winter wheat based on drip irrigation and sprinkler irrigation has shown remarkable water-saving effects by measuring soil moisture in real-time to guide crop irrigation [1,2,3]. However, due to the uneven distribution of soil moisture in the field and the lag of soil moisture monitoring, the traditional monitoring method of soil moisture lacks the ability to quickly, accurately and timely assess the crop water condition, and fails to meet the needs of guiding the precision irrigation of crops [4]. The real-time monitoring and scientific diagnosis of crop water status are the prerequisites for the timely and accurate irrigation of crops [5]. Although plant physiological indexes such as plant water content and leaf water potential are good indicators to reflect crop water status, they are relatively time-consuming in the measurement process, and the influence of spatial distribution differences of plant individuals and sample number limitations during field sampling leads to large errors in the measurement results [6,7]. Therefore, it is of great significance to choose scientific crop water monitoring methods for the timely and accurate diagnosis of crop water deficit and reasonable irrigation decisions.

Crop canopy temperature is an important indicator of the water status of plant populations [8]. With the advent of thermal imaging cameras, it provides a reliable means for quickly and easily obtaining canopy temperature information and the timely and accurate diagnosis of crop moisture status [9]. The remote sensing monitoring of canopy temperature to diagnose crop water status overcomes the limitation of traditional methods to represent the overall population with individual indicators, and it realizes the rapid and accurate determination of plant population water status in a large range, becoming a superior crop water diagnosis method than the determination of soil moisture and plant physiological indicators [10]. However, when using infrared thermal imaging cameras to extract canopy temperature, it is not only affected by environmental factors such as solar radiation, wind speed, air temperature and humidity, but also by the plant’s own physiological factors [11,12]. During the growth process of wheat, the environment is complex and changeable, and various environmental factors interact to affect the crop canopy temperature [13]. In addition, winter wheat has different drought tolerances at different growth stages, resulting in different sensitivities of plant physiological indicators (such as plant water content, Gs and Tr) to soil moisture [14]. These physiological indexes can not only reflect crop water status but also directly affect crop canopy temperature. Therefore, when using the canopy temperature information obtained by infrared thermography to diagnose crop water status, it is necessary to analyze the sensitivity of each physiological index to soil moisture and the correlation between canopy temperature information and various physiological indexes, to scientifically and accurately determine the water deficit of crops at each growth stage.

In this study, based on the analysis of different adaptation characteristics of plant physiological indexes to soil water at different growth stages of winter wheat, correlation analysis was conducted between the canopy temperature information obtained by infrared thermal imaging and soil water and plant physiological indexes, to study the effectiveness of canopy temperature information based on the infrared thermal image to diagnose crop water status. On the basis of the above research, canopy temperature information, biomass, yield and other indicators in different growth stages of winter wheat were comprehensively analyzed to determine the threshold range of suitable water for plants based on canopy temperature information in each growth stage, to guide crop irrigation.

## 2. Results

### 2.1. Characteristics of Soil Moisture and Yield under Different Treatments

**Figure 1:**
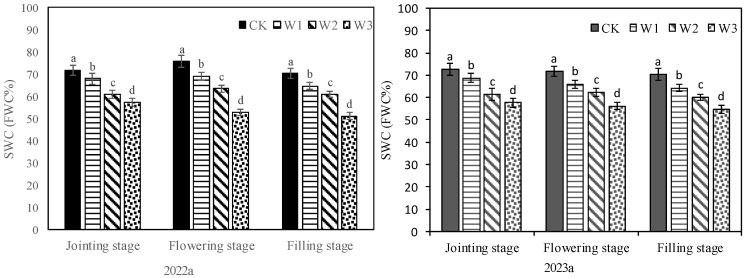
Soil water content (SWC) under different treatments. Different letters in the same growing stage show significant differences among treatments (*p* < 0.05). CK: adequate irrigation; W1: moderate irrigation; W2: slight deficit irrigation; W3: severe deficit irrigation.

Different irrigation treatments significantly affected the soil water status, and there were significant differences in SWC among different treatments at jointing, flowering and filling stages (Figure 1). The biomass and grain yield of crops were also affected by irrigation amounts. At the jointing stage, there was no significant difference in biomass among W1, W2 and CK, while the biomass of W3 was significantly reduced compared with that of CK (Table 1). In the two test years, the SWC of W3 at this stage was 57.4% FWC (2022a) and 57.6% FWC (2023a), respectively. At the flowering stage, the biomass of W1 was similar to that of CK, but the biomass of W2 and W3 decreased significantly compared with that of CK. In the two test years, the SWC of W2 at this stage was 63.4% FWC (2022a) and 62.3% FWC (2023a), respectively. At maturity, there was no significant difference in grain yield between W1 and CK, while the grain yield of W2 and W3 decreased significantly compared with that of CK. In the two test years, the SWC of W2 at the filling stage was 60.9% FWC (2022a) and 60.1% FWC (2023a), respectively. In addition, W1 had a higher harvest index compared with CK. Different irrigation treatments also significantly affected irrigation water use efficiency (IWUE) by affecting crop yield. The grain yield of W1 was not significantly different from that of CK, but it reduced the amount of irrigation water and therefore improved IWUE. The results of soil water and biomass (grain yield) in the two years showed that the plant production would not suffer from drought stress as long as SWC was maintained above 57.0% FWC during the jointing stage, 63.0% FWC during the flowering stage and 60.0% FWC during the filling stage.

### 2.2. Stomatal Behavior and Water Status of Plants under Different Treatments

**Figure 2:**
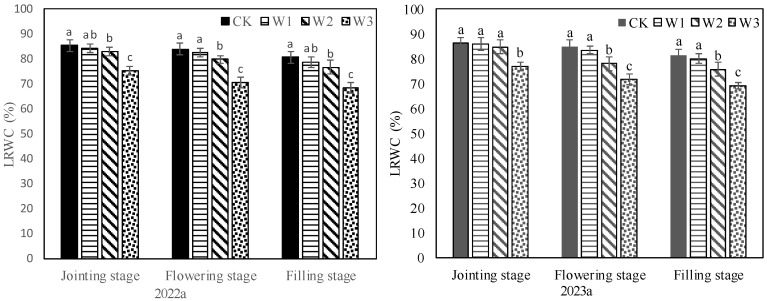
LRWC of plants under different treatments. Different letters in the same growing stage show significant differences among treatments (*p* < 0.05). CK: adequate irrigation; W1: moderate irrigation; W2: slight deficit irrigation; W3: severe deficit irrigation.

**Figure 3:**
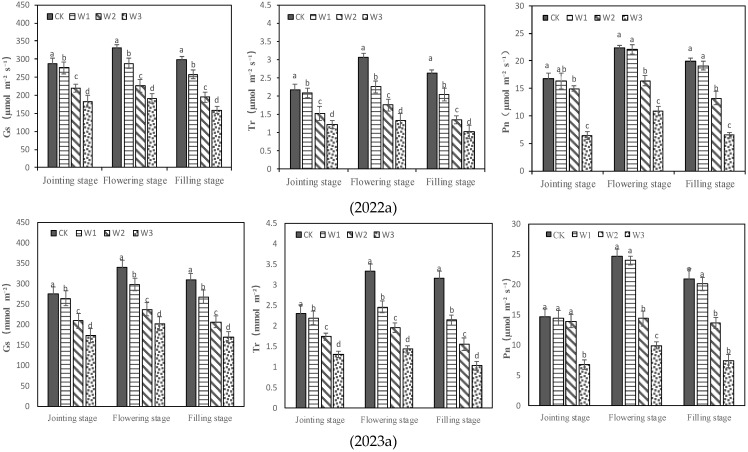
Stomatal behavior of plants under different treatments. Different letters in the same growing stage show significant differences among treatments (*p* < 0.05). CK: adequate irrigation; W1: moderate irrigation; W2: slight deficit irrigation; W3: severe deficit irrigation.

Different irrigation treatments had different effects on plant water status by affecting water absorption by the roots. At the jointing stage, the LRWC of W1 and W2 was the same as that of CK and higher than that of W3. At the flowering and filling stages, the LRWC of W1 was the same as that of CK, those of W2 and W3 were significantly lower than that of CK, and W3 had the lowest LRWC (Figure 2). Irrigation has an important effect on the stomatal behavior of plants by influencing their water status. At the jointing stage, the Gs and Tr of W1 were the same as those of CK, but those of W2 and W3 were lower than those of CK. At the flowering stage and filling stage, the Gs and Tr of plants in W1, W2 and W3 were lower compared to those of CK (Figure 3). The Pn of plants was different from Gs and Tr. At the jointing stage, the Pn in W1 and W2 was similar to that in CK, but that in W3 was significantly lower than that in CK. There was no significant difference in Pn between W1 and CK at the flowering stage and filling stage, but the Pn of W2 was lower than that of CK, and W3 had the lowest Pn. It can be seen that plant physiological parameters have various sensitivities to soil water in different growth stages, and the sensitivity of Gs and Tr to soil water was higher than that of LRWC and Pn.

### 2.3. Correlation Analysis of CWSI with SWC, LRWC and Stomatal Characteristic Parameters

**Figure 4:**
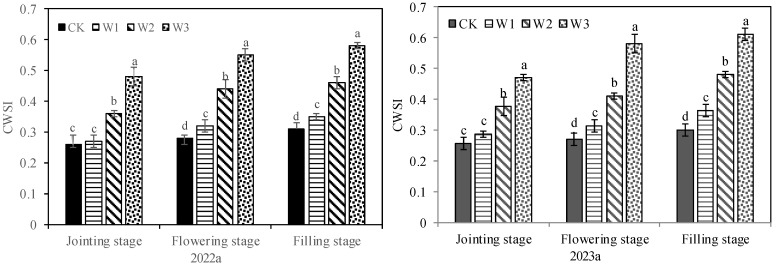
CWSI of plants under different treatments. Different letters in the same growing stage show significant differences among treatments (*p* < 0.05). CK: adequate irrigation; W1: moderate irrigation; W2: slight deficit irrigation; W3: severe deficit irrigation.

The CWSI of crops showed an increasing trend with the decrease in irrigation water (Figure 4). However, it is important to note that the CWSI of crops under different treatments exhibited distinct characteristics during the three test periods. At the jointing stage, the CWSI of W1 was similar to that of CK, and the CWSI of W2 and W3 was significantly greater than that of CK. At the flowering and filling stages, the CWSI of W1, W2 and W3 was significantly higher than that of CK, and W3 had the highest CWSI, followed by W2. Correlation analysis showed that the correlation of CWSI with Gs, Tr and Pn was lower than that with LRWC and SWC at the jointing stage (Table 2). CWSI was extremely significantly negatively correlated with SWC and LRWC (*p* < 0.01), but significantly negatively correlated with Gs, Tr and Pn (*p* < 0.05). At the flowering stage, CWSI was extremely significantly negatively correlated with SWC, Gs, Pn, Tr and LRWC (*p* < 0.01). At the filling stage, CWSI was extremely significantly negatively correlated with SWC, Gs, Tr and LRWC (*p* < 0.01), but significantly negatively correlated with Gs, Tr and Pn (*p* < 0.05).

### 2.4. Regression Analysis of CWSI with Biomass and Grain Yield

**Figure 5:**
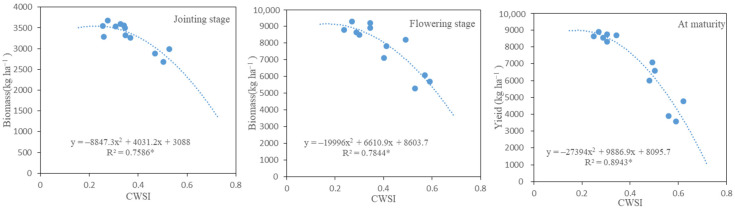
The relationship between CWSI and biomass and yield at different growth stages (2023). * indicates significant correlation at *p*< 0.05 levels.

Regression analysis showed that the CWSI of winter wheat at each growth stage was correlated with biomass (grain yield) in a curvilinear relationship (Figure 5). With the increase in CWSI, the biomass and yield of winter wheat did not change significantly in the early stage. When the CWSI increased to a certain extent, the biomass and grain yield showed a decreasing trend with the increase in CWSI. The R^2^ values of CWSI and biomass (grain yield) in the three growth stages were 0.7586, 0.7844 and 0.8943, respectively, and they were all significantly correlated (*p* < 0.05), indicating that there were significant quadratic function changes between CWSI and biomass (grain yield). In addition, according to the performance characteristics of plant physiology (Figure 2 and Figure 3), biomass/grain yield (Table 1) and CWSI (Figure 4) at each growth stage, there was no significant difference in biomass among WI, W2 and CK at the jointing stage, and the CWSI value of CK, W1 and W2 at this stage was 0.26, 0.27 and 0.36 in 2022, and 0.26, 0.29 and 0.38 in 2023, respectively. There was no significant difference in biomass between W1 and CK from the heading to flowering stage, and the CWSI value of CK and W1 at this stage was 0.28 and 0.32 in 2022, and 0.27 and 0.30 in 2023, respectively. At the maturity stage, the yield of W1 was similar to CK, and the CWSI value of CK and W1 during the filling stage was 0.31 and 0.35 in 2022, and 0.30 and 0.36 in 2023, respectively. Based on the above results, it can be concluded that crop production will not be affected by water stress as long as the CWSI value of crops is maintained at 0.26–0.38 during the jointing stage, 0.27–0.32 during the flowering stage and 0.30–0.36 during the filling stage.

## 3. Discussion

### 3.1. Sensitivity of Physiological Factors to Soil Water

Optimizing irrigation systems according to the sensitivity of crops to water shortage and the allowable water deficit at different growth stages has important guiding significance for the efficient utilization of agricultural water resources [15]. Under field conditions, crops have different requirements for suitable soil water due to differences in genotype, growth stage, soil conditions, etc. [16], which results in different sensitivities of physiological indicators reflecting plant water status to soil water. In this study, under the same water condition, wheat had different physiological characteristics at different growth stages. At the jointing stage, Gs and Tr of W1 and W2 were significantly lower than those of the control, but Pn, LRWC and biomass did not decrease significantly, indicating that the plants were not subjected to water stress. At the flowering stage and filling stage, the Pn, LRWC and biomass of plants in W2 were significantly decreased compared with the control, indicating that plants were under water stress. It can be seen that the adaptability of wheat physiological characteristics (such as LRWC, Gs, Tr and Pn) to soil water was also different in different growth stages. Monitoring only soil moisture cannot be used to accurately judge the true water status of crops, and soil moisture and plant physiological and ecological characteristics must be comprehensively analyzed in order to make scientific irrigation decisions. In addition, although the plant Gs and Tr of W1 decreased significantly during flowering, its Pn was not affected. This is because there is a linear relationship between plant Tr and Gs, while the relationship between Pn and Gs is gradually saturated [17]. The proper reduction in Gs has no significant effect on Pn, but can significantly reduce Tr, which helps to reduce plant water consumption and delay water stress, thus improving crop water use efficiency.

### 3.2. Sensitivity of Canopy Temperature to Soil Water and Plant Physiological Factors

The accurate diagnosis of plant water status is the premise to ensure timely and scientific crop irrigation. Although canopy temperature can reflect the water status of the crop population, it is often interfered with by external conditions (such as solar net radiation, wind speed, air temperature and humidity), thus affecting the accuracy of diagnosing crop water status by measuring canopy temperature [18]. Idso et al. [19] proposed the crop water stress index (CWSI) and defined an empirical model for CWSI calculation on the basis of considering the influence of air humidity and temperature. However, the CWSI calculation of this model does not take the real leaf as the reference surface. Jones [20] and Leinonen et al. [21] suggest using leaves sprayed with water from both sides to simulate full transpiration as a wet reference surface estimate, and using leaves coated with Vaseline on both sides to simulate leaves with no transpiration at all as a dry reference surface estimate. Canopy temperature was extracted by infrared thermal image technology, and CWSI was calculated to estimate the water stress of plants, which reduced the experimental error, and the calculated CWSI value could better evaluate the water status of crops. Previous studies have shown that the CWSI calculated by this method has a good correlation with SWC, Pn, Gs, leaf water potential and other indicators reflecting plant water status, which can better reflect the water status of crops [22,23]. Yuan et al. [24] proved that CWSI was closely related to leaf water potential, stomata resistance and soil water, and was an ideal index for monitoring the water status of winter wheat. At present, CWSI is a widely used indicator to reflect the water status of plants [25]. However, due to the long growth period of winter wheat, its environment is complex and changeable during the growth process of wheat, the plant’s own factors (such as its physiological and ecological characteristics) are also constantly changing, and the interaction of these factors jointly affects the crop canopy temperature [13]. For example, in this study, the sensitivity of plant physiological indexes (such as LRWC, Gs and Pn) to soil water was different at various growth stages, resulting in different characteristics of canopy temperature information (CWSI) at different growth stages. Therefore, when using canopy temperature to diagnose crop water status, the difference in drought tolerance at different growth stages of crops should also be considered, and the sensitivity of canopy temperature to crop physiological characteristics and soil water should be analyzed.

### 3.3. Feasibility Analysis of CWSI in Guiding Irrigation

Soil water status is the basis of irrigation decision making. Studying the relationship between CWSI and SWC, irrigation amount and crop production can provide a theoretical basis for using canopy temperature information to guide crop irrigation. There is a good consistency between CWSI and soil water change; CWSI can reflect the trend of farmland water change [26]. This study also showed that CWSI was significantly correlated with SWC, which could reflect plant water status and could be used as an effective index to evaluate crop water status. However, some studies suggest that using plant water status as the basis for irrigation decision making is more reliable than soil water status; especially, LRWC is the best indicator to reflect the degree of plant water surplus and deficit [27]. This study also showed that LRWC was significantly correlated with CWSI, and CWSI could be used to evaluate crop water status and guide crop irrigation. Previous studies have shown that wheat yield varies parabolically with irrigation amount, and an increasing irrigation amount within a certain range can improve the grain yield of wheat [28,29]. In this study, by analyzing the relationship between CWSI and yield, it is considered that CWSI and yield are significantly negatively correlated and conform to the parabolic equation. Therefore, CWSI can be used as a decision-making index to guide the water-saving irrigation of winter wheat. Developing a suitable irrigation scheduling according to the response of crops to water deficit and the lower limit of SWC at different growth stages can achieve the purpose of saving water and increasing the grain yield of crops [15]. There are significant differences in climatic conditions and plant adaptability to the environment at different growth stages of winter wheat, which should be considered when using canopy temperature information to guide crop irrigation [30]. In this study, by analyzing the change rules of CWSI, biomass (yield) and other indicators of crops and their correlation, it was found that the threshold range of CWSI for the water deficit in wheat at different growth stages was different. Therefore, it was necessary to pay attention to the differences in different growth stages of crops when using CWSI to guide irrigation. In addition, the CWSI critical value of crop water shortage in this study is somewhat different from those of Chen et al. [30] and Zhang et al. [31]. In addition to the fact that different soil backgrounds, canopy temperature measurement methods and meteorological factors of different determination times will affect the appropriate CWSI of crops, the different plant varieties used in this study are also reasons for the inconsistency between the results of this study and the conclusions of previous studies. Previous studies have shown significant differences in canopy temperature among wheat varieties [32]. Most of the relevant research results come from specific environments and plant varieties, which cannot play a broad role in guiding irrigation decisions of different crops under various environmental conditions. Therefore, further application research is needed on how to avoid the interference of background information such as environmental factors and plant varieties on canopy temperature and to select a scientific canopy temperature measurement method, to accurately obtain the canopy temperature information of crops.

## 4. Materials and Methods

### 4.1. Experimental Design

**Figure 6:**
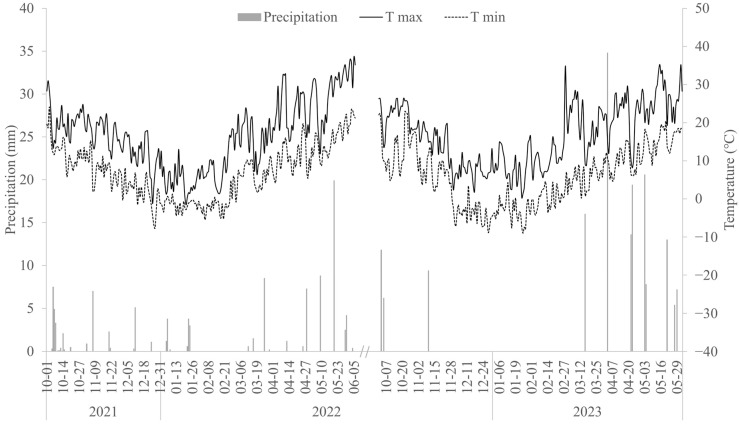
The meteorological information during the growing seasons. Tmax: maximum temperature; Tmin: minimum temperature.

This study was conducted from October 2021 to June 2023 in the experimental field of Henan Polytechnic University (35°11′ N, 113°15′ E). The meteorological data of each growing season are shown in Figure 6. The soil in the experimental field was clay loam. In the tillage layer (0–30 cm), the soil bulk density was 1.30 g·cm^−3^, the field water capacity (FWC) was 27.2% and the contents of total nitrogen (N), phosphorus and organic matter were 1.18 g kg^−1^, 0.92 g kg^−1^, 26.8 g kg ^−1^, respectively. The winter wheat variety used in this study was “Ping ’an 11”, which is widely cultivated in this area. The basal fertilizers consisting of N (120 kg ha^−1^), P (60 kg ha^−1^) and K (48 kg ha^−1^) were applied before wheat sowing (18 October). The experiment involved water treatments: full irrigation (CK), moderate irrigation (W1), slight deficit irrigation (W2) and severe deficit irrigation (W3). Each water treatment was irrigated according to the drip irrigation quantity designed in Table 3. Each treatment consisted of 3 replicated plots, and each plot had an area of 12 m^2^ (2.4 m × 5.0 m).

### 4.2. Monitoring Items and Methods

① Acquisition of infrared thermal images. During the jointing (16 March) and flowering stages (26 April) of wheat, infrared thermal images of wheat canopy were taken vertically at a distance of 50 cm above the canopy with an infrared thermal imager (Infrec G100, NEC, Tokyo, Japan) from 11:00 to 12:00 p.m. on a sunny day, and randomly shot 3 times per plot.

② Determination of dry and wet reference surface temperatures. According to the method of Leinonen et al. [21], two adjacent, equally sized and fully unfolded pest-free leaves were selected in the wheat canopy before canopy temperature measurement with an infrared thermal imager, and both sides of one leaf were evenly coated with Vaseline to close stomata as a dry reference surface. Water was simultaneously sprayed on both sides of the other leaf to keep it moist, as a wet reference surface. After the wet and dry reference surfaces were made, the temperature of the reference surface was stabilized for 1 min, and then the wheat canopy was photographed by infrared thermal imager. After taking photos, an infrared thermal image and the corresponding physical picture could be generated at the same time. In the supporting software, these two pictures could be opened at the same time, the position of the dry and wet reference surface in the infrared thermal image could be determined according to the physical picture, and the temperature of the dry and wet reference surface (Td, Tw, respectively) could also be obtained.

③ Calculation of canopy temperature. The image analysis software of the infrared thermal imager (Infrec Analyzer NS9500 Standard, S7.1E) was used to export all pixels in the thermal image into an Excel table, the temperature between the dry and wet reference surfaces was extracted, and its average value was used as the average temperature of the wheat canopy. This method can effectively remove the interference of soil, sky and other factors.

④ Soil and plant index determination.

The photosynthetic parameters: These were measured in sync with canopy temperatures. At the jointing and flowering stages, the Gs, Pn and Tr of the fully developed leaves were measured using an LI-6800 Portable Photosynthesis System (LICor, Inc., Lincoln, NE, USA) from 9:00 a.m. to 11:00. Each treatment was repeated 3 times.

Leaf relative water content (LRWC). After the photosynthetic parameters were determined, the same leaf was immediately collected to measure its LRWC, and each treatment was repeated 3 times.

Soil moisture measurement. After the photosynthetic parameters were determined, the soil moisture content of each treatment was measured by a soil moisture meter at 20 cm intervals. Soil moisture content was measured 0–60 cm at the jointing stage and 0–100 cm at the flowering stage.

### 4.3. Calculation of Crop Water Stress Index (CWSI)

The *CWSI* of crops was calculated according to the empirical formula of Jones [33]:

(1) $$CWSI=\frac{T_{c}-T_{w}}{T_{d}-T_{w}}$$

where *T_c_* is the temperature of the canopy leaf (°C); *T_d_* is the leaf temperature (°C) when the leaf stomata is closed and no transpiration occurs. *T_w_* is the leaf temperature (°C) at which the stomata of the leaves are open to the maximum and the leaves are in the full transpiration state. *T_d_* and *T_w_* were replaced by leaf temperatures where Vaseline was applied and water was sprayed, respectively.

### 4.4. Yield Traits and Irrigation Water Use Efficiency (IWUE)

At maturity (1 June), plants of 1.0 m^2^ were randomly collected from each plot to measure grain yield. IWUE was also calculated according to the following formula:

IWUE (kg ha^−1^mm^−1^) = Y (kg ha^−1^)/IR (mm) (2)

where Y is grain yield and IR is the recorded irrigation volume throughout the growing season.

### 4.5. Statistical Analysis

SPSS 25.0 software was used for statistical analysis and the differences among different treatments were compared using the least-significant-difference (LSD) tests (*p* < 0.05).

## 5. Conclusions

The sensitivity of plant physiological parameters to soil water was different at different growth stages. The sensitivity of Gs and Tr to soil water was higher than that of LRWC and Pn. The characteristics of plant physiology and biomass (yield) at each growth stage indicate that plant production will be protected from drought stress as long as soil moisture is maintained above 57.0% FWC during the jointing stage, 63.0% FWC during the flowering stage and 60.0% FWC during the filling stage. Correlation analysis showed that CWSI was significantly negatively correlated with all SWC and plant physiological parameters. The regression analysis revealed a curvilinear relationship between the CWSI of winter wheat and biomass (yield). As CWSI increased beyond a certain threshold, there was a corresponding decrease in both biomass and grain yield. Comprehensive analysis of all the indexes showed that CWSI can be used as a decision-making index to guide the water-saving irrigation of winter wheat. In order to avoid the adverse impact of water stress on crops and achieve water-saving production, the CWSI threshold of crops should be maintained at 0.26–0.38 during the jointing stage, 0.27–0.32 during the flowering stage and 0.30–0.36 during the filling stage.

## Figures and Tables

**Table 1:** Biomass, yield and IWUE of different treatments.

Treatments	Biomass at Jointing Stage (kg ha^−1^)		Biomass at Flowering Stage (kg ha^−1^)		Grain Yield (kg ha^−1^)		Harvest Index		Total Irrigation Water (mm)		IWUE (kg ha^−1^ mm^−1^)	
	2022	2023	2022	2023	2022	2023	2022	2023	2022	2023	2022	2023
CK	3622.5a	3501.4a	8951.2a	8916.8a	8601.3a	8692.4a	0.39b	0.40b	300	300	30.3b	30.0b
W1	3597.6a	3483.6a	8917.8a	8866.5a	8488.4a	8586.8a	0.43a	0.44a	240	240	36.9a	37.3a
W2	3584.9a	3444.7a	7438.6b	7700.7b	6501.7b	6565.1b	0.40b	0.41b	180	180	36.1a	36.5a
W3	3045.3b	2854.2b	5856.3c	5685.7c	4319.3c	4086.0c	0.39b	0.37c	160	160	27.0c	25.5c

Different letters in the same year imply significant differences between different treatments at *p* < 0.05. IWUE: irrigation water use efficiency. CK: adequate irrigation; W1: moderate irrigation; W2: slight deficit irrigation; W3: severe deficit irrigation.

**Table 2:** Correlation analysis of CWSI with SWC, LRWC and stomatal characteristic parameters.

	SWC	LRWC	Gs	Tr	Pn
Jointing stage	−0.6925 **	−0.6627 **	−0.5028 *	−0.5009 *	−0.5032 *
Flowering stage	−0.6768 **	−0.7549 **	−0.7340 **	−0.7177 **	−0.6084 **
Filling stage	−0.7018 **	−0.7197 **	−0.6620 **	−0.6326 **	−0.5081 *

* and ** indicate significant correlation at *p* < 0.05 and *p* < 0.01 levels, respectively.

**Table 3:** Different water treatments and their irrigation amounts (mm).

Treatments	Seedling Stage	Jointing Stage	Heading Stage	Filling Stage	Total
CK	80	80	80	60	300
W1	70	70	70	30	240
W2	60	60	60		180
W3	60	50	50		160

## Data Availability

Data will be made available on request.
